# Medical Treatment of Aortic Aneurysms in Marfan Syndrome and other Heritable Conditions

**DOI:** 10.2174/1573403X1002140506124902

**Published:** 2014-05

**Authors:** Christine H. Attenhofer Jost, Matthias Greutmann, Heidi M. Connolly, Roland Weber, Marianne Rohrbach, Angela Oxenius, Oliver Kretschmar, Thomas F. Luscher, Gabor Matyas

**Affiliations:** 1 From the Cardiovascular Center Klinik Im Park, Zurich, Switzerland;; 2 The Department of Cardiology, Congenital Heart Disease Division, University Heart Center, Zurich, Switzerland;; 3 The Division of Cardiovascular Diseases, Mayo Clinic, Rochester MN, USA;; 4 The Division of Cardiology, University Children’s Hospital, Zurich, Switzerland;; 5 The Division of Metabolism, University Children’s Hospital, Zurich, Switzerland;; 6 Center for Cardiovascular Genetics and Gene Diagnostics, Schlieren, Switzerland

**Keywords:** Angiotensin II receptor antagonists, aortic aneurysm, beta-blockers, genetics, Marfan syndrome, medical management, risk factors, statins.

## Abstract

Thoracic aortic aneurysms can be triggered by genetic disorders such as Marfan syndrome (MFS) and related aortic diseases as well as by inflammatory disorders such as giant cell arteritis or atherosclerosis. In all these conditions, cardiovascular risk factors, such as systemic arterial hypertension, may contribute to faster rate of aneurysm progression. Optimal medical management to prevent progressive aortic dilatation and aortic dissection is unknown. β-blockers have been the mainstay of medical treatment for many years despite limited evidence of beneficial effects. Recently, losartan, an angiotensin II type I receptor antagonist (ARB), has shown promising results in a mouse model of MFS and subsequently in humans with MFS and hence is increasingly used. Several ongoing trials comparing losartan to β-blockers and/or placebo will better define the role of ARBs in the near future. In addition, other medications, such as statins and tetracyclines have demonstrated potential benefit in experimental aortic aneurysm studies. Given the advances in our understanding of molecular mechanisms triggering aortic dilatation and dissection, individualized management tailored to the underlying genetic defect may be on the horizon of individualized medicine. We anticipate that ongoing research will address the question whether such genotype/pathogenesis-driven treatments can replace current phenotype/syndrome-driven strategies and whether other forms of aortopathies should be treated similarly. In this work, we review currently used and promising medical treatment options for patients with heritable aortic aneurysmal disorders.

## INTRODUCTION

Aortic aneurysm, with its first description as the cause of death of King George II in 1760 [1] is defined as aortic dilatation of greater than 50% of the normal diameter for age and body surface area and occurs most commonly due to medial degeneration of a localized portion of the aorta [2]. Thoracic aortic aneurysms (TAAs) are less common than abdominal aortic aneurysms (AAAs). It is thought that genetic predisposition has a higher impact in TAA than in AAA. Collagen, elastin, and fibrillin are main components of the aortic media [3] besides smooth muscle cells, fibroblasts and mucoid ground substance. Cardiovascular risk factors such as smoking [4], dyslipidemia, and hypertension [5] may also contribute to aortic aneurysm formation. Genetic causes of aortic aneurysms include Marfan syndrome (MFS; caused by *FBN1* mutations) [6], Loeys-Dietz syndrome (LDS; associated with mutations in the genes *TGFBR1*, *TGFBR2*, *TGFB2*, and *SMAD3*), aneurysms-osteoarthritis syndrome (AOS; caused by *SMAD3* mutations), vascular Ehlers-Danlos syndrome (EDS IV; caused by *COL3A1* mutations), familial thoracic aortic aneurysm/dissection (FTAAD; associated with *ACTA2*, *MYH11*, and *MYLK* mutations), cutis laxa syndrome (CL; associated with *ELN* and *EFEMP2* mutations), aortic valve disease (AOVD1; caused by *NOTCH1* mutations), arterial tortuosity syndrome (ATS; caused by *SLC2A10* mutations), X-linked Alport syndrome (XLAS; caused by *COL4A5* mutations), and Turner syndrome (45,X) as well as other congenital heart malformations [7-9]. Rarely, aortic aneurysms (AA) have also been associated with mutations in the genes* COL1A1*, *COL1A2,* *MED12 *or* SMAD4* as well as medium-sized AAs with mutations in the genes *PLOD3*, *ENG*, *ACVRL1* or *NF1*.

Aortic aneurysms predispose to aortic dissection and/or rupture. The degree of aortic dilatation is an important risk factor related to these life-threatening conditions that account for most of the mortality associated with MFS [10]. The risk of aortic dissection and/or rupture increases significantly with increasing size and is estimated at 14% per year when the aortic size is >6.0 cm [11]. Some patients experience dissection or rupture at an aortic dimension less than 5.0 cm, particularly in some forms of inherited connective tissue disorders, such as LDS and EDS IV. Indeed, patients with *TGFBR1* or *TGFBR2* mutation have been shown to dissect with aortic diameters well below 5.0 cm [12]. Similarly, mutations in the genes *COL3A1*, *MYLK*, and *SMAD3* can result in aortic dissection and/or rupture with little to no aortic dilatation [7, 8, 13].

Thus, it is recognized that measurement of the aortic dimension alone, without taking the underlying disease etiology into consideration, is not adequate for risk stratification and decision making. Another option is using the aortic size index (ASI), which is the dimension of the ascending aorta indexed to body surface area. In a retrospective study, using this ASI measurement 3 risk groups were identified. In Group 1, in which the aorta is less than 2.75 cm/m2, risk of aortic dissection or rupture is low, estimated at 4% per year. In Group 2 with aortic dimension from 2.75 to 4.24 cm/m2 the risk is moderate at approximately 8% per year, and in Group 3, in which the aorta measures more than 4.24 cm/m2, the risk is high (approximately 20% per year) [14]. However, the ASI values cited in this study are derived from a distinct population of TAA patients including - but certainly not limited to - MFS patients. Patients with congenital aortic malformations, such as coarctation of the aorta, were not included in this study.

The pathogenesis of aneurysm formation is multifactorial and includes medial degeneration, an inflammatory reaction, cellular proliferation/matrix degradation, and shear stress [15]. An important role has been attributed to reactive oxygen species which may activate matrix metalloproteinases (MMPs) [16], angiotensin II, cyclophilin A, TGF-β, osteoprotegerin, and tumor necrosis factors. The mitogen-actived protein kinase (MAPK)/extracellular signal-regulated kinase (ERK) cascade has recently also been implicated in aneurysm formation [15]. Accordingly, inhibition of the pathway MAPK/ERK cascade with statins and ERK inhibitors has reduced aneurysm formation in experimental models [17, 18]. Similarly, increased TGF-β signaling in the arterial wall has recently been shown as a common pathological feature in MFS, LDS, FTAAD, and AOS, explaining and emphasizing the beneficial effects of TGF-β signaling inhibitors on aortic dilatation [19]. A schematic overview of signaling pathways including TGFBR1, TGFBR2, TGFB2, and SMAD3 (canonical TGF-β signaling) as well as ERK1/2 (non-canonical TGF-β signaling) is shown in Fig. (**1**).

Emergent surgical repair is the only current treatment option for ascending aortic dissection. Elective surgical aortic replacement is recommended to prevent dissection when the ascending aorta is markedly enlarged. Medical treatment ideally prevents or slows progressive aortic enlargement and reduces the risk of associated life-threatening complications. Thus, the main goal of medical treatment for aortopathies is to reduce structural changes within the aortic wall and to prevent aortic dilatation. These medical treatments will likely be most effective when given in an early stage of the disease. Lowering systemic blood pressure below 120-130 mmHg and decreasing cardiac contractility are also thought to be beneficial strategies to prevent progressive aortic dilatation. Although aortic wall stiffness may be increased in MFS [20] and other conditions such as bicuspid aortic valve (BAV) and FTAAD, stiffer aortas do not appear to be associated with increased aneurysm growth rate [21]. Whether medical treatment reducing aortic wall stiffness affects outcomes needs to be determined.

Several medical treatments have been studied for prevention or delay of aortic dilatation. These include β-blockers, angiotensin converting enzyme inhibitors (ACEIs), angiotensin II type I receptor (AGTR1) blockers (ARBs), statins, tetracyclines/macrolides, and ERK inhibitors. As turkeys tend to develop aortic dissection at the age of 7-24 weeks, the first medical therapeutic studies on aortic dissection prevention were done in this animal model and subsequently in humans. In randomized controlled trials, the antipsychotic and antihypertensive drug reserpine was shown to reduce mortality. Because reserpine may lead to depression and Parkinson disease in men, it is now rarely used in the management of systemic hypertension in humans. In this work, we will review currently used and promising medical treatment options for patients with heritable aortic aneurysmal disorders.

## β-BLOCKER THERAPY

For decades β-blockers have been the mainstay of medical treatment of aortic aneurysms. Historically, β-blockers have been shown to reduce aortic aneurysm growth rate in turkey and human studies [22]. Although there is no significant change in central aortic pressure induced by β-blockers, a negative inotropic effect of β-blockers may reduce the amplitude of aortic wave reflections [23] and some improvement in elastic aortic properties has also been shown by the β-blocker atenolol.

Initially, efficacy of the β-blocker propranolol in decreasing the rate of aortic dilatation stems from a small randomized study of patients with MFS. Patients treated with propranolol (sample size = 32) had a 73% lower rate of aortic dilatation and lower mortality than placebo-treated patients [24]. In a subsequent randomized trial, propranolol was not found to significantly affect the growth rate of small AAAs [25]. Leach *et al*. [26] treated 12 patients with AAAs larger than 3 cm with β-blockers and compared to 15 untreated patients with no aneurysms, showing that the mean aortic diameter growth rate was significantly lower in patients taking β-blockers (0.17 cm/year versus 0.44 cm/year).

Some have suggested that ARBs and ACEIs may be more effective in maintaining aortic size in at-risk patients than β-blockers, however, this is still a matter of ongoing research and debate [27]. Although β-blockers may cause fatigue, erectile dysfunction, decrease in physical ability, depression, and an increase in body weight, which may adversely impact quality of life, β-blockers have been recommended in MFS patients with aortic dilatation and may be considered in patients with BAV and an aortic root diameter of >4 cm. However, a large meta-analysis did not show that β-blockers reduce mortality or the incidence of aortic dissection in patients with MFS [28].

Despite limited evidence for their efficacy, β-blockers are still widely used as first line therapy with an aim of preventing progression of aortic aneurysms in MFS and other disorders. The dosage of β-blockers should be adjusted depending on their effect. The heart rate after submaximal exercise should be<100 beats per minute. According to the ACC/AHA/AATS guidelines, β-blockers should be administered to all patients with MFS and aortic aneurysms unless contraindicated [29]. Hence, we currently add β-blockers in every patient with MFS and other conditions with a comparable or higher risk for aortic dissection such as LDS or AOS – especially in the presence of aortic dilatation with a z-score >2 and/or a positive family history for aortic dissection. In contrast, we do not routinely give β-blockers to normotensive patients with aortic dilatation in association with congenital heart disease such as BAV or tetralogy of Fallot.

## ANGIOTENSIN II TYPE I RECEPTOR BLOCKERS

ARBs are currently a major source of optimism in the treatment and prevention of TAAs and, thus, may be more often used than β-blockers in the disease management of MFS. In 2006, the beneficial effect of the ARB losartan on aneurysm progression was demonstrated in a mouse model of MFS [30]. MFS mice showed increased TGF-β signaling which was prevented by the administration of both TGF-β-neutralizing antibodies and prenatal losartan. Similarly, losartan given to MFS mice at the age of 6 weeks improved elastic fiber organization, increased aortic breaking stress, improved the contractile function of the aorta and reduced MMP activation. The endothelial nitric oxide (NO) pathway, however, remained suppressed in the thoracic aorta, which might limit the long-term benefits of losartan in MFS [31, 32]. If given in MFS mouse model prenatally, losartan prevented elastic fiber fragmentation in the aortic media; if given postnatally, it improved the life span but did not affect aortic vessel wall structure. Evidence for the efficacy of losartan in humans stems from Brooke *et al*. in 18 young patients with MFS [32]. In these initial case series, the rate of aortic root enlargement decreased considerably when comparing the time period prior and after the initiation of treatment with losartan. 

Meanwhile, nearly a dozen trials with different designs and inclusion criteria have started or are being planned around the world in order to determine the effect of ARBs in MFS (e.g. Gambarin *et al*., 2009 [33]; Detaint *et al*., 2010 [34]; Radonic *et al*., 2010 [35]; Möberg *et al*., 2012 [36], s. Table **1**). The next few years will therefore reveal whether ARBs will become the preferred therapeutic agent in MFS patients. Unfortunately, the trials use different ARBs (sartans), different drugs for comparison, and subjects of different ages. First results of the trials have recently been reported whereby NTR1423 (Table **1**) showed that treatment with losartan reduced the dilatation of the aortic root and – in patients after aortic root replacement – the growth of the aortic arch [37]. There was no significant difference in the need for prophylactic surgery and the study was not powered to detect an effect on the risk of aortic dissection or cardiovascular death. These findings have recently been supported by a smaller prospective study in 28 young patients with MFS (mean age 13.1 years, NCT00651235 in Table **1**), showing that the addition of losartan to a β-blocker (atenolol or propranolol) in 15 patients versus β-blocker only in 13 patients reduced the rate of aortic dilation significantly [38].

ACEIs inhibit the production of angiotensin II and thus reduce the signaling that occurs through both angiotensin II receptors (AGTR1 and AGTR2). There are no data on proving better long-term outcome with ARBs than ACEIs in patients with aneurysms. In a population-based case-control study from Canada involving patients with non-ruptured aortic aneurysms, patients receiving ACEIs had a decreased risk of AAA rupture compared to those who did not [39]. This effect was also seen in patients older than 75 years and in those with a history of hypertension [39].

On the basis of existing evidence, ACEIs and ARBs may have more beneficial effects than β-blockers on the progression of aortic dilation in MFS [27]. Furthermore, losartan has proved to be superior to ACEI in protecting the structure of the aortic wall and preventing aortic root dilation in MFS by blocking TGF-β mediated activation of ERK and preserving the beneficial angiotensin II type II receptor (AGTR2) pathway. However, in some patients ACEIs (dual AGTR1 and AGTR2 blockade) may nonetheless be more beneficial than ARBs (AGTR1 blockade) [40]. Importantly, ARBs and ACEIs are contraindicated during pregnancy due to teratogenicity. 

Summarizing the available evidence, currently the treatment of TAA patients with a combination of an ARB plus a β-blocker might offer the best protection of aortic dilatation. Further data from randomized trials will become available in the near future and may elucidate whether ARB or ACEI will not only reduce aortic dilatation but may also affect the risk of aortic dissection and mortality in MFS patients [27]. 

## CALCIUM CHANNEL BLOCKERS

There are limited data on the efficacy of CCBs in MFS. In a randomized, double-blind cross-over trial in 14 patients with MFS, the effect of the β-blocker atenolol, the ACEI perindopril and the calcium channel blocker (CCB) verapamil were compared [41]. The impact on peripheral and central systolic blood pressure in this study was equal, only atenolol slowed the heart rate and delayed aortic wave travel [41]. There may be some concerns with the use of CCBs in patients with MFS. At the American Heart Association Meeting 2012 data were presented showing that CCBs exacerbated aortic disease and caused premature lethality in MFS mice due to increased ERK activation [42]. Therefore, CCBs have to be used with caution in patients with MFS.

## STATINS

Statins (3-hydroxy-3-methylglutaryl coenzyme A reductase inhibitors) are primarily used to reduce cholesterol levels and thus reduce the progression of atherosclerosis through their lipid-lowering as well as through their so-called pleiotropic effects, and are one of the cornerstones in treatment of atherosclerotic disease. Due to their ability to reduce the expression of MMPs [43], there is the hope that statins may be helpful in the prevention of aortic dilatation as well. Reduction of the expansion rate of aortic aneurysms by statins has been reported in AAA, however in these AAA studies the aneurysms were still small in size [17, 44, 45]. Goel *et al*. reported that in 147 patients with BAVs the ascending aortic size was lower in the statin-treated group (76 patients) compared to the control group (71 patients) [46]. Multivariate analysis demonstrated statin use as the only independent factor that impacted aortic size and was even associated with a 0.33-cm reduction in aortic size (95% confidence interval 0.06 to 0.59, p <0.01) [46]. In a MFS mouse model with heterozygous *Fbn1* mutation, mice were treated daily from the age of 6 weeks with pravastatin or losartan. Both, pravastatin and losartan resulted in a significant reduction in aortic root dilatation and both preserved elastin volume in the medial layer [47].

Although generally safe, statins can cause some adverse effects, such as hepatic dysfunction, myalgias, and rarely rhabdomyolysis. Given the lack of solid efficacy data, current guidelines do not recommend the use of statins for patients with aortopathies in the absence of dyslipidemia.

## TETRACYCLINES/MACROLIDES

**Fig. (1):**
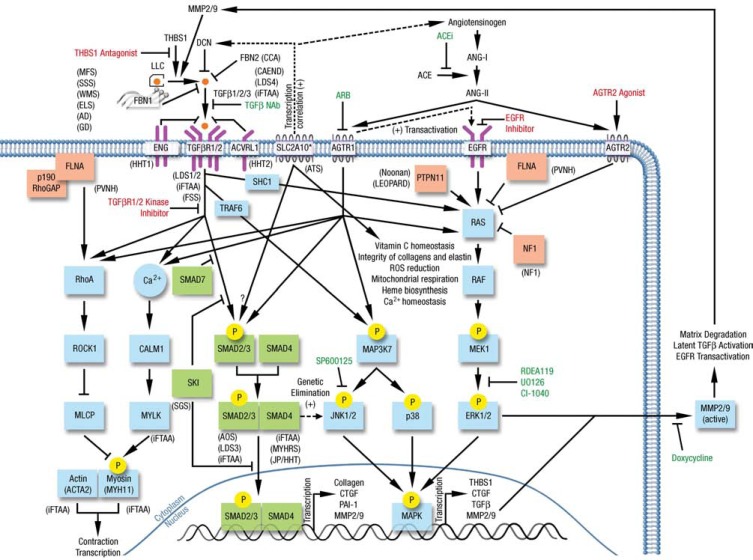
Canonical (green) and non-canonical (blue) TGF-β signaling cascades as well as intracellular proteins implicated in the TGF-β signaling (red) of Marfan syndrome and related disorders (adapted and modified from Doyle *et al.* [69], Lee *et al.* [70], and Willaert *et al.* [71]). Disorders caused by a mutated gene product are indicated in parentheses next to the corresponding protein. Drugs have been tested in Marfan mice and/or patients (green) and untested but may have hypothetical benefit based on disease pathogenesis (red) are illustrated accordingly. MFS: Marfan syndrome; SSS: Stiff skin syndrome; WMS: Weill-Marchesani syndrome; ELS: Ectopia lentis syndrome; AD: Acromelic dysplasia; GD: Geophysic dysplasia; CCA: Congenital contractural arachnodactyly; CAEND: Camurati-Engelmann disease; HHT1: Hereditary hemorrhagic telangiectasia type 1; HHT2: Hereditary hemorrhagic telangiectasia type 2; LDS1/2/3/4: Loeys Dietz syndrome types 1, 2, 3, 4; ATS: Arterial tortuosity syndrome; PVNH: Periventricular nodular heterotopia; iFTAA: Isolated familial thoracic aortic aneurysm; FSS: Ferguson Smith syndrome; SGS: Shprintzen-Goldberg syndrome; AOS: Aneurysms-osteoarthritis syndrome; MYHRS: Myhre syndrome; JP/HHT: Juvenile polyposis/hereditary hemorrhagic telangiectasia syndrome. NAb: neutralizing antibody; ARB: Angiotensin II type I receptor blocker; ACEi: Angiotensin converting enzyme inhibitor; SLC2A10*: SLC2A10/GLUT10 is mainly localized to the mitochondria of aortic smooth muscle cells.

The activation of TGF-β leads to upregulation of MMPs (cf. Fig. **1**), which increase aneurysm expansion. Doxycycline, a tetracycline, has been shown to delay aneurysm rupture in a mouse model [48]. Doxycycline-treated MFS mice lived longer compared to untreated mice and had decreased elastic fiber degradation paralleling lower MMP-2 and MMP-9 levels. There are also studies with the macrolide roxithromycin: 84 patients were randomized to an annual 4-week treatment with roxithromycin or placebo and those with roxithromycin had less progression of small AAAs and delayed need for surgical repair [49]. However, currently these results cannot be extrapolated to TAAs and even less so to congenital aortopathies.

Of note, some data suggest that there might be gender differences. In one study, examining patients with AAAs, women had higher levels of MMP-9 compared to men with equally large AAAs. One might speculate that MMPs may serve as a biomarker related to the sex differences in aneurysm development [50]. 

## ESTROGENS

Some of the differences between genders may be attributed to estradiol as well. Treatment with estradiol has been shown to prevent AAA development by inhibiting proteolytic activity in the aortic wall [51]. Ethinyl estradiol is occasionally given to girls with MFS to limit somatic growth [52]. It is possible that this treatment may have a beneficial impact on aortic growth rate. However, it has not been established that estrogen deficiency, which is frequently observed in patients with Turner syndrome, increases the risk of aortic dissection.

## ERK INHIBITORS

ERKs (ERK1 and ERK2) are involved in cell signaling that may cause aortic aneurysms and dissections. It has been demonstrated in the aorta of fibrillin-1 deficient mice that TGF-β- and AGTR1-dependent activation of ERKs is involved in TAA and that pathological aortic root growth can be abrogated by treatment with a specific ERK inhibitor [18, 53]. In contrast to ACEIs, losartan can inhibit TGF-β mediated ERK activation by allowing continued signaling through AGTR2, which is protective. If losartan treatment is without beneficial effect, ERK inhibitors as well as AGTR2 agonists could help, but these novel treatment options need further experimental validation in appropriate mouse models before translation to patients can be realized.

## ONGOING STUDIES 

Most of the pertinent questions will only be answered by the ongoing and future studies comparing currently used therapies and novel approaches. Some of the ongoing trials are summarized in Table **1**. The largest ongoing trial is funded by the NIH and organized by the Pediatric Heart Network, comparing. the effect of atenolol versus losartan in young patients with MFS and aortic dilatation [54]. Another ongoing study is the Ghent Marfan Trial, a randomized, double-blind placebo controlled trial with losartan versus placebo in patients already treated with β-blockers aiming to enroll 174 patients, aged ≥10 years and with an aortic z-score ≥2 [36]. The patients will be followed for 3 years at 6 monthly intervals [36]. In an Italian study, Gambarin *et al*. will include 291 patients with MFS and proven *FBN1* mutations with aortic root dilatation and a z-score of ≥2.5 in an open label phase 3 study [33]. They will compare losartan and the β-blocker nebivolol or a combination of both on the progression of aortic growth rate [33]. This study will have the advantage of a study focused on patients with proven *FBN1* mutation. A French research team is studying 300 patients at least 10-year-old fulfilling the Ghent criteria for MFS [34]. The effect of losartan will be assessed in a multicenter, randomized, placebo-controlled, double-blinded trial. Patients will be enrolled for 2 years with a 3-year-follow-up. Forteza *et al*. [55] are conducting a double-blind trial with 150 patients (5-60-year-old) with MFS according to the Ghent criteria comparing atenolol versus losartan.

We eagerly anticipate the results of these studies which will shed additional insight on the most pressing questions we have regarding medical treatment in our patients with aortopathies. It will be important to elucidate whether treatment effects are best when agents are given early prior to aortic dilatation or if these agents remain effective in MFS patients with dilated aortas.

## CARDIOVASCULAR RISK FACTORS AND SLEEP APNEA

Smoking induces an increased collagenase activity in human aortic aneurysm walls, particularly in larger or ruptured aneurysms. Smokers have an increased elastolytic activity in polymorphonuclear leukocytes [56]. Hypertension is also a risk factor for aneurysm rupture based on the law of Laplace. It is important to achieve good blood pressure control in all patients with aortic aneurysms. Obesity is also quite common in patients with MFS, an increased body mass index is found in 36% of these patients [57]. Overall, it is essential that patients with MFS or related connective tissue disorders and patients with aortopathy in association with congenital heart disease have optimal control of all cardiovascular risk factors. Recommendations for blood pressure control in patients with connective tissue disorders advise a systolic blood pressure of<130 mmHg in MFS/LDS and<120 mmHg in EDS IV, being applicable to other aortopathies as well (see emergency guidelines at www.orpha.net). In addition, the incidence of obstructive sleep apnea is increased in MFS and has been reported to occur in about 30% of such cases [58]. Furthermore, sleep apnea can increase the incidence of hypertension, atrial fibrillation, and heart failure in MFS patients.

## AORTIC ANEURYSMS IN CONGENITAL HEART DISEASE

Aortopathy in various forms of congenital heart disease has been reported. Aortic aneurysms occur frequently in patients with BAV [59], aortic coarctation [60], conotruncal abnormalities [61], and Turner syndrome [62]. Many of the latter patients also have BAV and/or coarctation. It is currently not known how many patients with BAV have mutation in the *TGFBR1*, *TGFBR2* or *FBN1* gene. There are only few reported cases of patients with aortic aneurysm in the setting of BAV with known genetic defects. However, there is also evidence that hemodynamic perturbations also play a role in the development of aortopathy in patients with BAV [63]. Besides, there is a genotype-phenotype correlation: it was recently reported that patients with BAV and prominent valve calcification and dysfunction with a low penetrance of aortic aneurysm are more likely to have *NOTCH1* mutation compared to those with non-calcified BAV and highly penetrant aortic aneurysms [64]. Although overall the risk of aortic dissection in patients with BAV is low, over a 25-year period, in a study by the Mayo Clinic, there was a 25% risk of aortic surgery and 26% risk of aortic aneurysm formation [65]. Even in the absence of data from randomized trials, it may be considered to use a β-blockers and/or an ARB to slow aortic root growth in BAV patients. Unfortunately, there are no data on medical prevention of aortic dilatation in coarctation, conotruncal abnormalities, and patients with Turner syndrome.

## MEDICAL TREATMENT ADJUSTED TO THE UNDERLYING GENETIC DEFECT

As reviewed above, patients with aortic aneurysm of different etiologies may respond differently to medical treatment. For example, whereas patients with mutations leading to increased level of TGF-β could benefit from a treatment with the ARB drug losartan, patients with mutations leading to increased proteolysis in the aortic wall may respond to a drug therapy with the MMP inhibitor doxycycline (cf. Fig. **1**). Currently, patients with *FBN1* mutation can benefit from the combination therapy of losartan and β-blocker. In the future, not only the mutated gene itself but also the nature of mutation could be of therapeutic importance. Accordingly, patients with heterozygous mutations leading to true or functional haploinsufficiency (i.e. one allele loses functionality) could benefit from medical treatments that increase expression of the normal allele and/or enable the read-through of premature termination codons in the case of functional haploinsufficiency due to nonsense-mediated mRNA decay. Thus, one may hope in the future that by elucidating the underlying genetic defect the most appropriate medical treatment can be targeted to each individual patient. By using high throughput targeted, whole-exome or whole-genome sequencing, the technical ease with which to determine a genetic mutation has increased. The gene defects usually sought in patients with a thoracic aneurysm are shown in Table **2** and include *FBN1*, *TGFBR1*, *TGFBR2*, *TGFB2*, *SMAD3*, *ACTA2*, *MHY11*, and *MYLK*. Notably, the identification of the underlying genetic defect is very important for the most appropriate surveillance and management of affected relatives as well.

## CURRENT RECOMMENDATIONS FOR OPERATIVE AORTIC ROOT REPLACEMENT

Guidelines for aortic root replacement depend on different factors such as the underlying etiology of aortic aneurysm and associated valvular heart disease, planned pregnancy, family history of aortic dissection, and aortic growth. In Table **3**, some of the current recommendations for aortic root surgery are summarized. Apart from the actual size of the aortic diameter, also the rate of change is important for decision-making in terms of prophylactic operation and the frequency of follow-up. It is important to be aware of the different ways and recommendations to measure aortic size (e.g. inner edge to inner edge versus leading edge to leading edge; echocardiography versus cardiac magnetic resonance). 

## CONCLUSION

Within the last two decades, knowledge of the genetic causes and therapeutic targets for patients with the propensity to develop an aortic aneurysm has grown substantially. However, the optimal choice of medical treatment is still largely unknown. The ongoing prospective trials, including double-blind comparison of β-blockers versus losartan should provide some clarification regarding the optimal therapy in certain patient groups in the near future. It appears that TGF-β mediated ERK activation emerges as the predominant driver of aneurysm progression and that the beneficial effect of losartan and ERK-specific inhibitors depends on the ERK signaling status. However, large-scale controlled studies are required to confirm this beneficial effect.

In the current era, all patients with thoracic aortic aneurysms require multidisciplinary care which should include meticulous monitoring of aortic dimension by non-invasive imaging, optimization of cardiovascular risk factors, screening of first degree relatives and prophylactic operative intervention. It appears that the majority of patients will also benefit from initiation of medical therapy with β-blocker and/or ARB, especially if the aortic aneurysm is genetically triggered. We anticipate the day that each aortic aneurysm patient will receive a specific genetic diagnosis and individualized therapy specific for that diagnosis.

## Figures and Tables

**Table 1.** Overview of clinical trials on pharmacological treatment of dilatation of the aorta in Marfan syndrome (ARB, β-blocker or both).

Trial identifier (reference)	Year trial started	Goal (study design)	Number of patients, time follow-up	Age group included	β-blocker (sample size), dosing	ARB (sample size), dosing	Outcome parameter
NCT00429364 (Lacro *et al*. [54])	2007	Comparing losartan vs. atenolol (randomized, single blind)	604 MFS patients, 3 years follow-up	6 months to 25 years and aortic root z-score >3.0	Atenolol (302), 0.5-4 mg/kg/day max. 250 mg/day	Losartan (302), 0.3-1.4 mg/kg/day max. 100 mg/day	Rate of change in aortic root BSA adjusted z-score
NCT00651235 (Chiu *et al*. [38]	2007	Efficacy of losartan added to β-blocker (randomized, open label)	28 MFS patients, 35 months of follow-up	≥1 year and aortic root z-score ≥2.0	β-blockers (13), atenolol or propranolol max. 150 mg/day for adults and 2 mg/kg/day for children	Losartan and β-blocker (15), 100 mg/day for adults and 50 mg/day for children	Change in aortic root diameter
NCT00782327 (MÖberg *et al*. [36])	2009	Additive effect of losartan and β-blocker (randomized, double blind)	174 MFS patients, 3 years follow-up	≥10 years and aortic root z-score ≥2	β-blocker and placebo (87), no data on dosing	Losartan and β-blocker (87), 25-50 mg/day below 50 kg or 50-100 mg/day over 50 kg	Decrease in aortic root growth rate
NCT00763893 (Detaint *et al*. [34])	2008	Efficacy of losartan vs. placebo (randomized, double blind)	300 MFS patients, 3 years follow-up	≥10 years	No β-blocker	Losartan (150) and placebo (150), 50 mg/day below 50 kg or 100 mg/day over 50 kg	Change in aortic root diameter
NTR1423 (Radonic *et al*. [35])	2008	Efficacy of losartan vs. not-treated controls (randomized, open label)	330 MFS patients, 3 years follow-up	≥18 years	No β-blockers (165) but patients continue taking their standard β-blocker treatment	Losartan (165), 50 mg/day (0-14 days) or 100 mg/day (>14 days)	Change in aortic root diameter and skin gene expression
NCT01145612 (Forteza *et al*. [55])	2008	Efficacy of losartan vs. atenolol (randomized, double blind)	150 MFS patients, 3 years follow-up	5-60 years and aortic diameter <45 mm	Atenolol (75), 12.5 mg/day (0-14 days) and 25 mg/day (>14 days) below 50 kg or 25 mg/day (0-14 days) and 50 mg/day (>14 days) over 50 kg	Losartan (75), 12.5 mg/day (0-14 days) and 25 mg/day (>14 days) below 50 kg or 25 mg/day (0-14 days) and 50 mg/day (>14 days) over 50 kg	Progression of dilation of the aortic valve annulus, sinuses of Valsalva, sinotubular junction, ascending aorta, aortic arch, thoracic and abdominal aorta
NCT00683124 (Gambarin *et al*. [33])	2008	Effects of losartan vs. nebivolol vs. the combination of both (randomized, open label)	291 MFS patients with FBN1 mutation, 4 years follow-up	12 months to 55 years and aortic root z-score ≥2.5 but <50 mm	Nebivolol (97 + 97 in combination?), max. 10 mg/day for adults and max. 0.16 mg/kg/day for children <16 years	Losartan (97 + 97 in combination?), max. 100 mg/day for adults and max. 1.6 mg/kg/day for children <16 years	BSA and age-adjusted aortic root diameter (sinuses of Valsalva), drug responsiveness (losartan: CYP2C9 gene, nebivolol: CYP2D6 gene)
NCT00723801 (ClinicalTrials.gov)	2007	Effects of losartan vs. atenolol on aortic stiffness (randomized, double blind)	50 MFS patients, 6 months follow-up	≥25 years	Atenolol (25?), 50 mg/day	Losartan (25?), 100 mg/day	Aortic biophysical properties and diastolic function

NCT, ClinicalTrials.gov Identifier; NTR, Netherlands trial register; MFS, Marfan syndrome

**Table 2.** Summary of diseases associated with an increased frequency of aortic aneurysm and dissection.

Disorder/syndrome	Inheritance	Prevalence (incidence)	Aortic aneurysm	Early aortic dissection	Arterial tortuosity	Other cardiovascular features	Gene (karyotype)	Pathway
Marfan syndrome	AD	~1:5,000	++	+	-	IA, MVP	*FBN1*	TGF-ß
TGFBR1/TGFBR2-related Loeys-Dietz syndrome (LDS) and thoracic aortic aneurysm/dissection (TAAD)	AD	unknown	++	+++	++	BAV, IA, MVP	*TGFBR1, TGFBR2 *	TGF-ß
SMAD3-related LDS, TAAD, and aneurysms-osteoarthritis syndrome	AD	unknown	++	++ / +++	++	BAV, IA, MVP	*SMAD3*	TGF-ß
TGFB2-related LDS and TAAD	AD	unknown	++ / +++	+	+	BAV, MVP	*TGFB2*	TGF-ß
ACTA2-, MYH11-, and MYLK-related TAAD	AD	unknown	+++	++	-	BAV, CAD (*ACTA2*), PDA	*ACTA2, MYH11, MYLK*	IGF-1, Ang II
Ehlers-Danlos syndrome, vascular type (EDS IV)	AD	~1:50,000	+	++ (rupture)	+	IA, MVP	*COL3A1*	collagen metabolism
Ehlers-Danlos syndrome, kyphoscoliotic form (EDS VIA)	AR	(~1:100,000)	+	++ (rupture)	-	MVP	*PLOD1*	collagen metabolism
PTPN11-related Noonan and LEOPARD syndromes	AD	~1:2,000	+	+	-	pulmonary valve stenosis	*PTPN11*	RAS-MEK-ERK
JAG1-related Alagille syndrome	AD	(1:70,000)	+	+	-	pulmonary valve stenosis, COA, IA, TOF	*JAG1*	NOTCH1-JAGGED1
Aortic valve disease	AD	unknown	+	+	-	BAV with valve calcification/dysfunction	*NOTCH1*	NOTCH1-JAGGED1
Congenital contractural arachnodactyly	AD	unknown	+	-	-	atrial/ventricular septal defects, MVP	*FBN2*	TGF- ß
SKI-related Shprintzen-Goldberg syndrome	AD	unknown	++	-	+	MVP, splenic artery aneurysm	*SKI*	TGF- ß
ELN-related cutis laxa	AD	(<1:4,000,000)	+	+	-	-	*ELN*	unknown
EFEMP2-related cutis laxa	AR	(<1:4,000,000)	++	+	++	arterial stenoses	*EFEMP2*	TGF-ß
Arterial tortuosity syndrome	AR	unknown	+	+	+++	arterial stenoses	*SLC2A10*	TGF-ß
FLNA-related periventricular heterotopia	XLD	unknown	+	+	-	BAV, PDA	*FLNA*	unknown
Fabry disease, cardiac variant	XL	(~1:3,000)	+	+	+	HCM	*GLA*	unknown
X-linked Alport syndrome	XL	(<1:50,000)	+	+	-	-	*COL4A5*	collagen metabolism
Turner syndrome	sporadic	(1:2,000)	+	++	-	BAV, COA, IA, LVOTO	(45,X)	unknown

BAV, bicuspid aortic valve; CAD, coronary artery disease; COA, coarctation of the aorta; HCM, hypertrophic cardiomyopathy; IA, intracranial aneurysms; LVOTO, left ventricular outflow tract obstruction; MVP, mitral valve prolapse; PDA, patent ductus arteriosus; TOF, tetralogy of Fallot; -, absent or not observed/reported; +, sporadic; ++, common; +++, typical; AD, autosomal dominant; AR, autosomal recessive; XL, X-linked; XLD, X-linked dominant.

**Table 3.** Current guidelines for operative aortic root replacement.

Disorder/syndrome	Aortic root replacement if size is larger than*	References
Marfan syndrome	5 cm	[6, 29]
TGFBR1/TGFBR2-related Loeys-Dietz syndrome (LDS) and thoracic aortic aneurysm/dissection (TAAD)	4-4.2 cm (internal diameter) or 4.4-4.6 cm CT and/or MRI	[29, 66]
SMAD3-related LDS, TAAD, and aneurysms-osteoarthritis syndrome; TGFB2-related LDS and TAAD	No data, may similar to LDS	
ACTA2-, MYH11-, and MYLK-related TAAD; Ehlers-Danlos syndrome, vascular type (EDS IV); Ehlers-Danlos syndrome, kyphoscoliotic form (EDS VIA); PTPN11-related Noonan and LEOPARD syndromes; JAG1-related Alagille syndrome	No data	
Turner syndrome	4.5-5 cm (aorta >2.5 cm/m2)	[67, 68]

* Comment: Earlier prior to pregnancy, positive family history for aortic dissection, rapid growth of the aorta (>5 mm/year), associated aortic valve disease, maximum aortic cross-sectional/area/body height >10 cm^2^/m.

## References

[1] Criado FJ ( 2011). Aortic dissection: A 250-year perspective. Texas Heart Institute journal / from the Texas Heart Institute of St. Luke's Episcopal Hosptal.Texas Children's Hospital..

[2] Johnston KW, Rutherford RB, Tilson MD, Shah DM, Hollier L, Stanley JC (1991). Suggested standards for reporting on arterial aneurysms.Subcommittee on Reporting Standards for Arterial Aneursms.ad hoc Committee on Reporting Standards., Society for Vascular Surgery and North American Chapter., International Society for Cardiovascular Surgery. J Vasc Surg.

[3] Coady MA, Rizzo JA, Goldstein LJ, Elefteriades JA ( 1999). Natural history. pathogensis.and etiology of thoracic aortic aneurysms and dissections. Cardiol Clin.

[4] Bonser RS, Pagano D, Lewis ME  (2000). Clinical and patho-anatomical factors affecting expansion of thoracic aortic aneurysms. Heart.

[5] Ito S, Akutsu K, Tamori Y  (2008). Differences in atherosclerotic profiles between patients with thoracic and abdominal aortic aneurysms. Am J Cardiol.

[6] Loeys BL, Dietz HC, Braverman AC  (2010). The revised Ghent nosology for the Marfan syndrome. J Med Genet.

[7] van der Linde D, van de Laar IM, Bertoli-Avella AM  (2012). Aggressive cardiovascular phenotype of aneurysms-osteoarthritis syndrome caused by pathogenic SMAD3 variants. J Am Coll Cardiol.

[8] van de Laar IM, Oldenburg RA, Pals G etal (2011). Mutations in SMAD3 cause a syndromic form of aortic aneurysms and dissections with early-onset osteoarthritis. Nat Genet..

[9] Plaisance BR, Winkler MA, Attili AK, Sorrell VL (2012). Congenital bicuspid aortic valve first presenting as an aortic aneurysm. Am J Med.

[10] Judge DP, Dietz HC (2005). Marfan's syndrome. Lancet.

[11] Elefteriades JA (2008). Thoracic aortic aneurysm: Reading the enemy's playbook. Yale J Biol Med..

[12] Yetman AT, Beroukhim RS, Ivy DD, Manchester D (2007). Importance of the clinical recognition of Loeys-Dietz syndrome in the neonatal period. Pediatrics.

[13] Wang L, Guo DC, Cao J  (2010). Mutations in myosin light chain kinase cause familial aortic dissections. Am J Human Genet..

[14] Gallo A, Davies RR, Coe MP, Elefteriades JA, Coady MA ( 2005). Indications. tiing.and prognosis of operative repair of aortic dissections. Semin Thorac Cardiovasc Surg.

[15] Danyi P, Elefteriades JA, Jovin IS (2012). Medical therapy of thoracic aortic aneurysms. Trends Cardiovasc Med.

[16] Longo GM, Xiong W, Greiner TC, Zhao Y, Fiotti N, Baxter BT (2002). Matrix metalloproteinases 2 and 9 work in concert to produce aortic aneurysms. J Clin Invest.

[17] Zhang Y, Naggar JC, Welzig CM  (2009). Simvastatin inhibits angiotensin II-induced abdominal aortic aneurysm formation in apolipoprotein e-knockout mice: Possible role of ERK. Arterioscler Thromb Vasc Biol.

[18] Habashi JP, Doyle JJ, Holm TM  (2011). Angiotensin II type 2 receptor signaling attenuates aortic aneurysm in mice through ERK antagonism. Science.

[19] Renard M, Callewaert B, Baetens M  (2013). Novel MYH11 and ACTA2 mutations reveal a role for enhanced TGFbeta signaling in FTAAD. Int J Cardiol.

[20] Baumgartner D, Baumgartner C, Schermer E  (2006). Different patterns of aortic wall elasticity in patients with Marfan syndrome: A noninvasive follow-up study. J Thorac Cardiovasc Surg.

[21] de Wit A, Vis K, Jeremy RW (2013). Aortic stiffness in heritable aortopathies: Relationship to aneurysm growth rate. Heart Lung Circ.

[22] Simpson CF, Kling JM, Palmer RF (1968). The use of propranolol for the protection of turkeys from the development of beta-aminopropionitrile-induced aortic ruptures. Angiology.

[23] Ohte N, Narita H, Sugawara M  (2003). Clinical usefulness of carotid arterial wave intensity in assessing left ventricular systolic and early diastolic performance. Heart Vessels.

[24] Shores J, Berger KR, Murphy EA, Pyeritz RE (1994). Progression of aortic dilatation and the benefit of long-term beta-adrenergic blockade in Marfan's syndrome. N Engl J Med.

[25] (2002). Propanolol Aneurysm Trial I.Propranolol for small abdominal aortic aneurysms: Results of a randomized trial. J Vasc Surg.

[26] Leach SD, Toole AL, Stern H, DeNatale RW, Tilson MD (1988). Effect of beta-adrenergic blockade on the growth rate of abdominal aortic aneurysms. Arch Surg.

[27] Thakur V, Rankin KN, Hartling L, Mackie AS (2013). A systematic review of the pharmacological management of aortic root dilation in Marfan syndrome. Cardiol Young.

[28] Gersony DR, McClaughlin MA, Jin Z, Gersony WM (2007). The effect of beta-blocker therapy on clinical outcome in patients with Marfan's syndrome: A meta-analysis. Int J Cardiol.

[29] Hiratzka LF, Bakris GL, Beckman JA  (2010). American College of Cardiology Foundation/American Heart Association Task Force on Practice G American Association for Thoracic S American College of R. American Stroe A.Society of Cardiovascular A Society for Cardiovascular A Interventions Society of Interventional R Society of Thoracic S Society for Vascular M. 2010 ACCF/AHA/AATS/ACR/ASA/SCA/SCAI/SIR/STS/SVM guidelines for the diagnosis and management of patients with thoracic aortic disease American College of Cardiology Foundation/American Heart Association Task Force on Practice Guidelines., American Association for Thoracic Surgery., American College of Radiology., American Stroke Association., Society of Cardiovascular Anesthesiologists., Society for Cardiovascular Angiography and Interventions., Society of Interventional Radiology., Society of Thoracic Surgeons., and Society for Vascular Medicine. Circulation.

[30] Habashi JP, Judge DP, Holm TM  ( 2006). Losartan. an AT1 antagoist.prevents aortic aneurysm in a mouse model of Marfan syndrome. Science.

[31] Yang HH, Kim JM, Chum E, van Breemen C, Chung AW (2009). Long-term effects of losartan on structure and function of the thoracic aorta in a mouse model of Marfan syndrome. Br J Pharmacol.

[32] Brooke BS, Habashi JP, Judge DP, Patel N, Loeys B, Dietz HC3rd (2008). Angiotensin II blockade and aortic-root dilation in Marfan's syndrome. N Engl J Med.

[33] Gambarin FI, Favalli V, Serio A  (2009). Rationale and design of a trial evaluating the effects of losartan vs.nebivolol vs. the association of both on the progression of aortic root dilation in Marfan syndrome with FBN1 gene mutations. J Cardiovasc Med.

[34] Detaint D, Aegerter P, Tubach F  (2010). Rationale and design of a randomized clinical trial (Marfan sartan) of angiotensin II receptor blocker therapy versus placebo in individuals with Marfan syndrome. Arch Cardiovasc Dis.

[35] Radonic T, de Witte P, Baars MJ  (2010). Losartan therapy in adults with Marfan syndrome: Study protocol of the multi-center randomized controlled compare trial. Trials.

[36] Moberg K, De Nobele S, Devos D  (2012). The Ghent Marfan trial - a randomized. double-blind placebo controlled trial with losartan in Marfan patients treated with beta-blockers. Int J Cardiol.

[37] Groenink M, den Hartog AW, Franken R  (2013). Losartan reduces aortic dilatation rate in adults with Marfan syndrome: A randomized controlled trial. Eur Heart J.

[38] Chiu HH, Wu MH, Wang JK  (2013). Losartan added to beta-blockade therapy for aortic root dilation in Marfan syndrome: A randomized. open-label pilot study. Mayo Clin Proc.

[39] Hackam DG, Thiruchelvam D, Redelmeier DA (2006). Angiotensin-converting enzyme inhibitors and aortic rupture: A population-based case-control study. Lancet.

[40] Danyi P, Jovin IS (2010). Is losartan the true panacea for aneurysm disease?. Con. Cardiol Clin.

[41] Williams A, Kenny D, Wilson D  (2012). Effects of atenolol. perindopril and verapamil on haemodynamic and vascular function in Marfan syndrome - a random sed.double-blind., crossover trial. Eur J Clin Invest.

[42] Doyle JJ, Habashi JP, Lindsay ME, Bedja D, Dietz HC (2010). Calcium channel blockers exacerbate aortic disease and cause premature lethality in Marfan syndrome. Circulation.

[43] Nagashima H, Aoka Y, Sakomura Y  ( 2002). A 3-hydroxy-3-methylglutaryl coenzyme a reductase inhibitor. cerivasttin.suppresses production of matrix metalloproteinase-9 in human abdominal aortic aneurysm wall. J Vasc Surg.

[44] Schouten O, van Laanen JH, Boersma E  (2006). Statins are associated with a reduced infrarenal abdominal aortic aneurysm growth. Eur J Vasc Endovasc Surg.

[45] Sukhija R, Aronow WS, Sandhu R, Kakar P, Babu S (2006). Mortality and size of abdominal aortic aneurysm at long-term follow-up of patients not treated surgically and treated with and without statins. Am J Cardiol.

[46] Goel SS, Tuzcu EM, Agarwal S  (2011). Comparison of ascending aortic size in patients with severe bicuspid aortic valve stenosis treated with versus without a statin drug. Am J Cardiol.

[47] McLoughlin D, McGuinness J, Byrne J  (2011). Pravastatin reduces Marfan aortic dilation. Circulation.

[48] Xiong W, Knispel RA, Dietz HC, Ramirez F, Baxter BT (2008). Doxycycline delays aneurysm rupture in a mouse model of Marfan syndrome. J Vasc Surg.

[49] Vammen S, Lindholt JS, Ostergaard L, Fasting H, Henneberg EW (2001). Randomized double-blind controlled trial of roxithromycin for prevention of abdominal aortic aneurysm expansion. Br J Surg.

[50] Villard C, Wagsater D, Swedenborg J, Eriksson P, Hultgren R (2012). Biomarkers for abdominal aortic aneurysms from a sex perspective. Gend Med.

[51] Hellenthal FA, Buurman WA, Wodzig WK, Schurink GW (2009). Biomarkers of AAA progression.Part 1: Extracellular matrix degeneration. Nat Rev Cardiol.

[52] Ucar SK, Paterson WF, Donaldson MD, Young D (2009). Ethinyl estradiol treatment for growth limitation in girls with Marfan's syndrome--experience from a single center. Endocr Res.

[53] Holm TM, Habashi JP, Doyle JJ  (2011). Noncanonical TGFbeta signaling contributes to aortic aneurysm progression in Marfan syndrome mice. Science.

[54] Lacro RV, Dietz HC, Wruck LM  (2007). Rationale and design of a randomized clinical trial of beta-blocker therapy (atenolol) versus angiotensin II receptor blocker therapy (losartan) in individuals with Marfan syndrome. Am Heart J.

[55] Forteza A, Evangelista A, Sanchez V  (2011). Study of the efficacy and safety of losartan versus atenolol for aortic dilation in patients with Marfan syndrome. Rev Esp Cardiol.

[56] Cannon DJ, Read RC (1982). Blood elastolytic activity in patients with aortic aneurysm. Ann Thorac Surg.

[57] Yetman AT, McCrindle BW (2010). The prevalence and clinical impact of obesity in adults with Marfan syndrome. Can J Cardiol.

[58] Kohler M, Blair E, Risby P  (2009). The prevalence of obstructive sleep apnoea and its association with aortic dilatation in Marfan's syndrome. Thorax.

[59] Siu SC, Silversides CK (2010). Bicuspid aortic valve disease. J Am Coll Cardiol.

[60] Bromberg BI, Beekman RH, Rocchini AP  (1989). Aortic aneurysm after patch aortoplasty repair of coarctation: A prospective analysis of prevalence. screening tests and risks.. J Am Coll Cardiol.

[61] Tan JL, Gatzoulis MA, Ho SY (2006). Aortic root disease in tetralogy of Fallot. Curr Opin Cardiol.

[62] Mortensen KH, Hjerrild BE, Stochholm K  (2011). Dilation of the ascending aorta in Turner syndrome - a prospective cardiovascular magnetic resonance study. J Cardiovasc Magn Reson.

[63] Kim YG, Sun BJ, Park GM  (2012). Aortopathy and bicuspid aortic valve: Haemodynamic burden is main contributor to aortic dilatation. Heart.

[64] Kent KC, Crenshaw ML, Goh DL, Dietz HC (2013). Genotype-phenotype correlation in patients with bicuspid aortic valve and aneurysm. J Thorac Cardiovasc Surg.

[65] Michelena HI, Khanna AD, Mahoney D  (2011). Incidence of aortic complications in patients with bicuspid aortic valves. JAMA.

[66] Paterick TE, Humphries JA, Ammar KA  (2013). Aortopathies: Etiologies. geneics.differential diagnosis., prognosis and management.. Am J Med.

[67] Matura LA, Ho VB, Rosing DR, Bondy CA (2007). Aortic dilatation and dissection in Turner syndrome. Circulation.

[68] Bondy CA (2008). Aortic dissection in Turner syndrome. Curr Opin Cardiol.

[69] Doyle JJ, Gerber EE, Dietz HC (2012). Matrix-dependent perturbation of TGFbeta signaling and disease. FEBS Lett.

[70] Lee YC, Huang HY, Chang CJ, Cheng CH, Chen YT (2010). Mitochondrial GLUT10 facilitates dehydroascorbic acid import and protects cells against oxidative stress: Mechanistic insight into arterial tortuosity syndrome. Hum Mol Genet.

[71] Willaert A, Khatri S, Callewaert BL  (2012). GLUT10 is required for the development of the cardiovascular system and the notochord and connects mitochondrial function to TGFbeta signaling. Hum Mol Genet.

